# Developing low-cost house floors to control tungiasis in Kenya – a feasibility study

**DOI:** 10.1186/s12889-023-17427-4

**Published:** 2023-12-12

**Authors:** Lynne Elson, Shadrack Mwadai Nyawa, Abneel Matharu, Ulrike Fillinger

**Affiliations:** 1 KEMRI-Wellcome Trust, Kilifi, Kenya; 2 Centre for Tropical Medicine and Global Health, Nuffield Department of Medicine, University of Oxford, Oxford, UK; 3 Dabaso Tujengane CBO, Watamu, Kenya; 4 Human Health Theme, International Centre for Insect Physiology and Ecology, Nairobi, Kenya

**Keywords:** Tungiasis, Tunga penetrans, Floors, Housing, NTD, Prevention

## Abstract

**Context:**

Tungiasis is a neglected tropical skin disease endemic in resource-poor communities. It is caused by the penetration of the female sand flea*, Tunga penetrans*, into the skin causing immense pain, itching, difficulty walking, sleeping and concentrating on school or work. Infection is associated with living in a house with unsealed earthen house floors.

**Methods:**

This feasibility study used a community-based co-creation approach to develop and test simple, locally appropriate, and affordable flooring solutions to create a sealed, washable floor for the prevention of tungiasis. Locally used techniques were explored and compared in small slab trials. The floor with best strength and lowest cost was pilot trialed in 12 households with tungiasis cases to assess its durability and costs, feasibility of installation in existing local houses using local masons and explore community perceptions. Disease outcomes were measured to estimate potential impact.

**Results:**

It was feasible to build the capacity of a community-based organization to conduct research, develop a low-cost floor and conduct a pilot trial. The optimal low-cost floor was stabilized local subsoil with cement at a 1:9 ratio, installed as a 5 cm depth slab. A sealed floor was associated with a lower mean infection intensity among infected children than in control households (aIRR 0.53, 95%CI 0.29–0.97) when adjusted for covariates. The cost of the new floor was US$3/m^2^ compared to $10 for a concrete floor. Beneficiaries reported the floor made their lives much easier, enabled them to keep clean and children to do their schoolwork and eat while sitting on the floor. Challenges encountered indicate future studies would need intensive mentoring of masons to ensure the floor is properly installed and households supervised to ensure the floor is properly cured.

**Conclusion:**

This study provided promising evidence that retrofitting simple cement-stabilised soil floors with locally available materials is a feasible option for tungiasis control and can be implemented through training of community-based organisations. Disease outcome data is promising and suggests that a definitive trial is warranted. Data generated will inform the design of a fully powered randomized trial combined with behaviour change communications.

**Trial registration:**

ISRCTN 62801024 (retrospective 07.07.2023).

**Supplementary Information:**

The online version contains supplementary material available at 10.1186/s12889-023-17427-4.

## Background

Tungiasis (known as ‘jiggers’ in Kenya), is a highly neglected parasitic skin disease, inflicting pain and suffering on millions of impoverished people in sub-Saharan Africa [1, 2], but has only recently been included in the World Health Organisation’s (WHO) list of neglected tropical diseases (NTD) [3]. Tungiasis is caused by the penetration of the female sand flea, *Tunga penetrans*, into the skin, mostly of the feet [4, 5]. The disease affects the most resource poor families primarily children and elderly people that depend on others for care [6–8]. Tungiasis is associated with a pattern of debilitating morbidity. Itching, pain, swelling, deep fissures, ulcers and abscess formation are symptoms of an acute inflammatory response to embedded fleas and bacterial superinfection of the lesions. Chronic infections result in chronic pain, disability, disfigurement, and mutilation of the feet [4, 9, 10]. Children with tungiasis are often ridiculed by their peers and it has been shown that both physical incapacity and mental strain and distress reduce school performance [11]. There is currently no effective, widely available, safe and simple treatment for tungiasis [2]. Instead affected individuals in their desperation remove the embedded fleas using unsterilized pins and thorns which carry huge risk of secondary infection with bacteria and viruses [12]. In resource-poor communities without optimal medical care and limited ability to pay for expensive medications, prevention is the most valuable control measure.

Multiple community-based risk factor surveys have identified a key determinant of the disease to be living in a house with poor construction characteristics, mud walls and unsealed soil floors [13–16]. A school-based survey of 1829 school children in coastal Kenya confirmed these results [17]. Whilst non-hardened classroom floors might provide an additional risk-factor for the disease, the house floor where the children slept was the leading factor in that study. Calculation of the population attributable fractions (PAF) suggested that the overall prevalence of tungiasis could be reduced by a third, and that of severe tungiasis by over a half, if sleeping places of children had sealed floors [17].

Sand fleas, like any other flea species [18], have environmental development stages. The immature off-host stages, develop on the ground, where they live and feed within the upper surface layer of loose, dry soil or sand [5]. However, information on the spatial distribution and abundance of off-host stages is absent for the African continent.. A pilot survey at the Kenyan coast and in eastern Uganda investigated soil samples taken from various locations inside and outside houses in homesteads affected by the disease (Matharu, Elson, Fillinger 2023, in prep.). This pilot survey revealed over 80% of the immature sand fleas extracted originated from indoor samples.

These direct entomological observations combined with the qualitative risk factor surveys strongly suggest that people’s houses are hotspots for sand flea development and continuous transmission. Based on this understanding, it is plausible to assume that disease burden can be reduced by hardening and sealing house floors. However, the standard procedure to improve floors for formal settlements includes layers of decreasing grades of rock ballast (hard core), a waterproof membrane and wire mesh (rebar), sealed with a top layer of concrete and/or tiling [19]. Such floors require formally trained masons for construction and costs for such standard floors have been estimated at a minimum of US$20/m^2^ in Kenya [19]. The average house size for a typical family affected by tungiasis in the study area is approximately 30 m^2^, thus a total cost of US$600, well beyond the reach of these families who survive on less than US$1 a day.

Here we present the results from a pump-priming project, which aimed at generating new transdisciplinary capabilities and to initiate novel research at the intersection of the built environment and health. Our objective was to explore end user perceptions on household flooring, develop a low-cost floor,and to estimate, its practical feasibility, potential challenges, floor costs, durability, and potential impact on disease outcomes such as prevalence, intensity of infection and associated pathology.

## Methods

### Study area and population

**Fig. 1:**
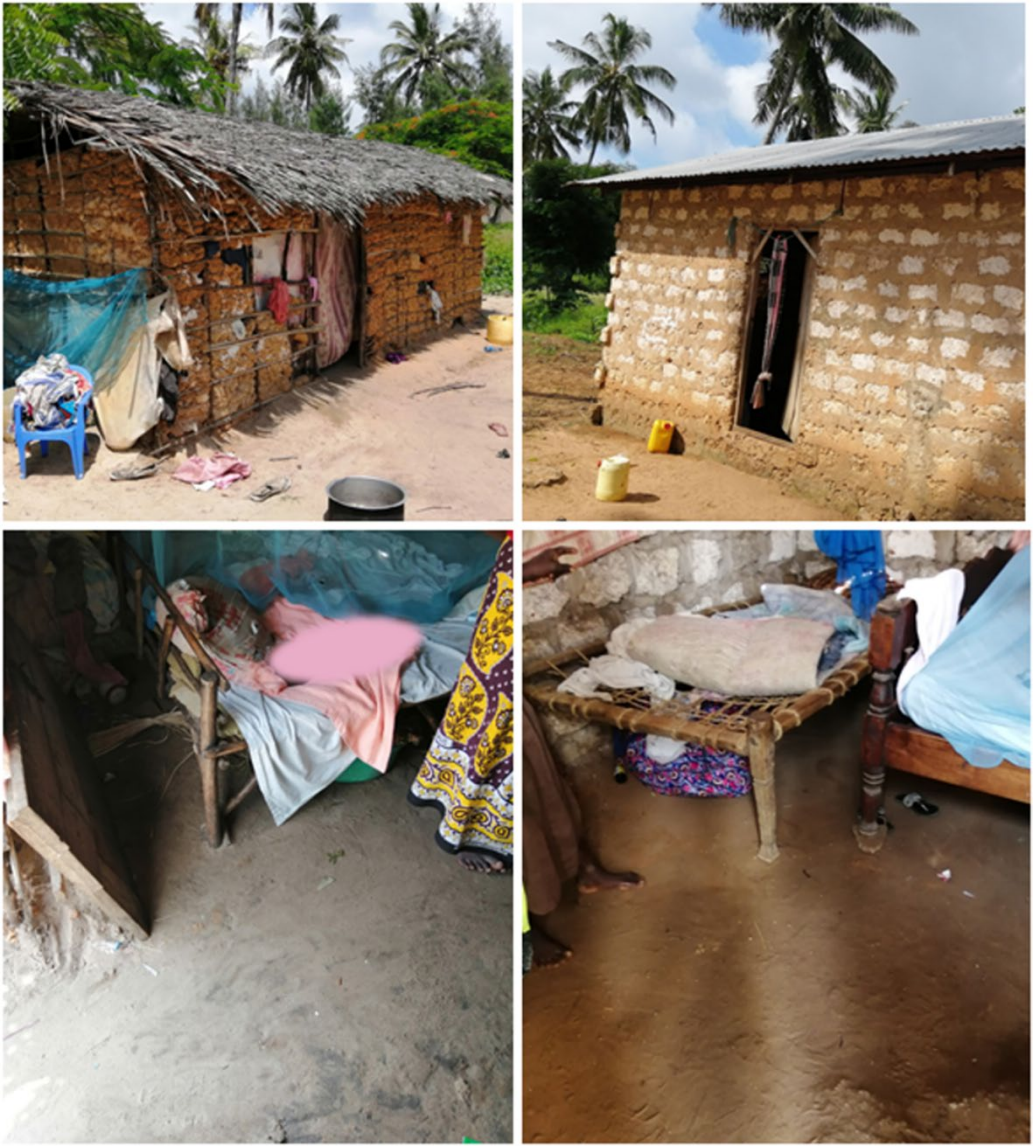
Typical rural mud and brick houses with their unsealed earthen floors in the study area in coastal Kenya

The study was conducted between April 2019 and October 2020 in rural settlements in the Watamu and Mida area (− 3.352521, 39.976521) 50 km north of Kilifi town in Kilifi County on the coast of Kenya. Here people live in mud walled houses with roofs of palm leaf thatch or corrugated iron sheets, or in houses with stone walls and iron sheet roof, but majority with an unsealed soil floor (Fig. 1). The population is almost uniformly of the Giriama ethnic group who rely on fishing in the sea and subsistence farming, or on income from activities related to tourism. Most families own chickens, ducks, cats, dogs and goats which roam freely, including inside the houses. The area has a hot humid climate with its main rainy seasons in April/May and November/December and a hot dry season from January to March. Since rainfall has been reported to impact tungiasis disease burden [20] the rainfall (mm) records were acquired for the time period of the project from World Weather Online [21] (Additional file 1)).

### Study approach

The study comprised three phases: 1) formative research in the target population to explore attitudes towards floors, as well as current and past methods of floor hardening, 2) characterizing the local soil and exploring different soil stabilization materials and methods that were locally appropriate, and 3) piloting a flooring intervention to test the feasibility of methods and procedures for later use on a large scale and to estimate possible impact on the disease and associations that might be worth to further investigate in a subsequent larger study.

To explore whether local primary health infrastructure and resources could be used to implement a flooring intervention, the study was conducted through a partnership with a community-based organization (CBO) and local masons. The CBO worked closely with the primary health care system and community health workers who normally provide preventive health services. They had several years of experience implementing a tungiasis control program in their community. Staff of the CBO were trained to identify suitable participants for each phase of the study following the study protocol and standard operating procedures. They identified local masons and worked with them to develop the low-cost floor and install floors for the trial.

### Formative focus group discussions

Six focus group discussions (FGDs); two with adult men and two with adult women (25–99 years), one with male youth (18–27 years) and one with female youth were implemented. The sessions were attended by 9–15 people who were identified by community health volunteers. The FGDs were facilitated by one of the authors (SMN) following a discussion guide and covered knowledge of floor types, reasons for sealing floors, methods and materials used currently and in the past in the community, floor cleaning and maintenance practices. The sessions were conducted in the local language, Giriama, recorded, transcribed, and translated to English.

### Floor development and testing

The aim was to find a low-cost but strong enough way to seal house floors in the study area. For this, 15 outdoor slabs, 1 m × 2 m, were constructed, as detailed in Table 1. The materials selected were based on local availability as well as on information received from the community during FGDs. The slabs were excavated to 15, 20 or 30 cm depths (foundation) and back filled with subsoil or hardcore to 10 or 15 cm depth (base layer), compacted and topped with the floor layer of 5–15 cm depth.

**Table 1:** Outdoor floor slab composition

	Depths (cm)		Composition of Top Layer (litres)														
Slab number	Base layer compressed	Top layer depth	Cement	Local Subsoil	Water	Building Sand	Coconut fibre compressed	Dabaso soil	Ant hill soil	Fire Ash	Cow dung	Lime	Alkali	Sodium Silicate	Caustic	small stones	Hardcore in base layer (cm)
1		15	45	–	30	225											15
2	15	15	45	225	30												
3	15	15	45	240	30												
4	10	10	22.5	150	–		1										
5	10	10	22.5	–	20			150									
6	10	5	15	–	15				56								
7	10	5	15	75	22					7.5							
8	10	5	22.5	150	10						15						
9	10	5	–	75	–							0.2	15	10.5	4.5		
10	10	5	–	75	–							5.3	15	10.5	4.5		
11	10	5	7.5	75	10												
12	10	5	7.5	75	20					7.5	7.5						
13	10	5	7.5	75	20							7.5					
14	10	5	15	75	20												
15	10	5	15	–	10	75										30	

The test floor layer comprised a range of materials including local site subsoil mixed with various other materials to bind the soil particles, prevent cracking and create a hard, smooth surface. These included: soil from a termite mound, fire ash, coconut fibres or cow dung, or a mix of lime, sodium hydroxide and silicate with or without cement at varying ratios (0–15%). Termite mound soil is very hard, containing termite enzymes which bind the soil particles close together and increases the compressive strength of mixes [22, 23]. Coconut fibre, and cow dung which contains grass fibres, help to bind the soil particles and prevent cracking [24]. Cow dung also contains enzymes which react with chemicals in the soil to bind the soil particles [24]. Fire ash contains calcium oxide and is sometimes used in the local community and has been shown to improve the strength and binding of soils [25, 26]. The silicate mix is a soil binder replacement for cement [27]. These floor slabs were compared to a hardcore and concrete slab (slab #1, Table 1) as a ‘positive control’ with 15 cm depth of hardcore rock, overlaid with builders’ sand purchased from a hardware merchant, mixed with 15% cement (15 cm depth) finished with a thin cement and water slurry later to fill cracks and uneven surfaces to obtain a smooth and level finish. Each slab containing cement was watered daily for 7 days to cure. All slabs were covered throughout with plastic sheeting to protect them from the weather.

After 8 and 28 days from construction, all slabs were tested for strength, wear and permeability using improvised testing equipment. Industrial instruments were not available in this rural setting, so tests were improvised. The load bearing strength was assessed using a wheelbarrow with 100 kg weight which was rolled across it 2 times. Impact strength was evaluated by dropping a weight of 5 kg from a height of 1.5 m onto the slab once. Resistance to abrasion was tested by dragging a piece of wood across the surface while applying pressure. Permeability was assessed by pouring 300 ml of water onto the surface whilst checking for the time of absorption. The impact of each test was scored on a scale of 0 to 3; 0 being no visible impact, 1 a little, 2 some, 3 a large impact. The impact of the wheelbarrow and weight drop were assessed as the depth of the mark created, if any. For the water absorption, 0 for no absorption, 3 for most of the water was absorbed. The day 8 and day 28 scores were summed up for evaluation at the end (the lower the score, the better the floor). The cost of each slab was calculated based on the locally procured material, transport and labour. The slab composition with the best strength, wear and permeability (lowest score) and lowest cost was chosen to install into houses in the pilot field trial.

### Pilot field trial

A small-scale pilot field trial was implemented to test improved floors under real-world conditions in people’s houses (retrospective trial registration ISRCTN 62801024 at 07.07.2023). A randomised controlled approach was pursued with houses being selected and randomized to one of three treatments: concrete floor, new low-cost floor and no floor. Note that the ‘concrete’ floor was a base layer of hardcore overlaid with concrete, but for simplicity is referred to throughout the manuscript as the ‘concrete floor’.

#### Floor installation

**Fig. 2:**
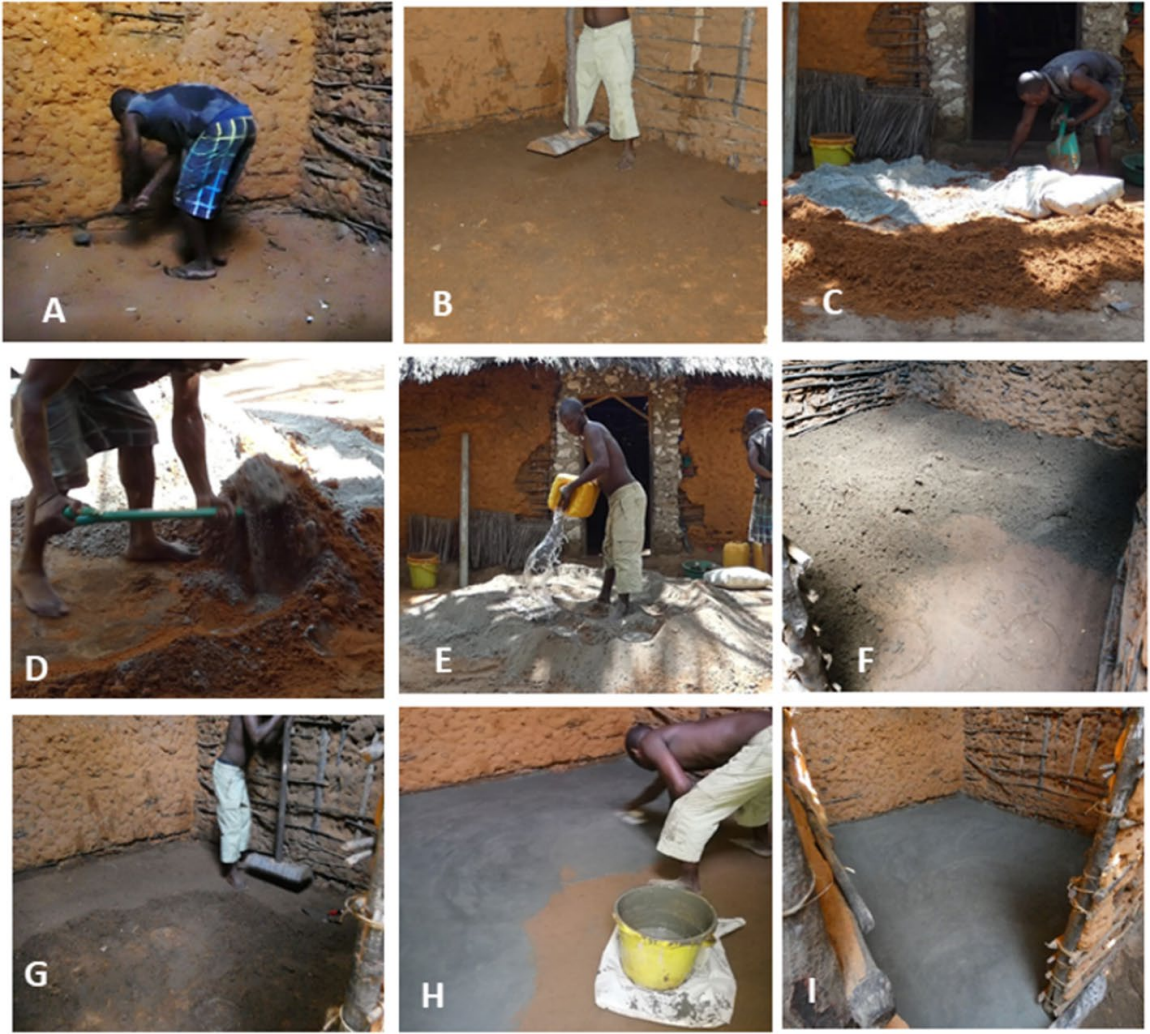
Installation of the low-cost floor. A) Excavating the existing surface, B) Compacting the soil, C) Adding cement to local subsoil (1 part cement with 9 parts soil), D) Mixing cement with sub-soil, E) Adding water to the mix, F) Cement-stabilized soil laid on top of the compacted soil, G) Compacting the cement-stabilized soil, H) Spreading the *“nill”* finish (cement/water slurry), I) The completed floor

The floors were installed on the day after the baseline survey. The CBO visited each household ahead of time to plan them moving their personal items out of the house and assisted them when necessary. Families were asked to find somewhere else to sleep for 7 days to allow the floor to cure. The floors were installed by a trained local mason (one for concrete floors and one for the low-cost floor), assisted by the labourer they usually employ to assist them. The family only provided the water that was needed for the construction and curing. The soil of the house floor was excavated to 15 cm depth (Fig. 2A), the soil redistributed, levelled and compacted (Fig. 2B). Subsoil from the family compound was collected, 30 l for every square metre of floor area. This was mixed thoroughly with cement at a 9:1 soil: cement ratio (Fig. 2C & D), and then water added at a 1:0.5 cement: water ratio (Fig. 2E). The amount of water varied slightly according to the moisture and clay content of the subsoil at each household. The soil mix was then spread on the floors (Fig. 2F) compacted to a depth of 5 cm levelled (Fig. 2G) and finished off with the cement slurry to give a smooth surface (Fig. 2H & I). The head of household or caregiver was shown how to sprinkle water every day for 7 days after installation for curing and the family asked not to move back into the house for 7 days. At the end of the study, all the control houses received the new low-cost floor.

#### Participants

Inclusion criteria for households were having an unsealed house floor, a house footprint of less than 40 m^2^ (for project budget reasons only), at least two family members infected with at least 5 fleas each, and the family willing and able to move out of the house while the floor was installed and cured. Eligible households were identified through a survey conducted in November 2019 by the local CBO and community health workers to identify households with tungiasis infected residents. The heads of eligible households were invited to a meeting where the study was explained, including the randomization process and controls. All heads of households who signed the consent form selected their own study arm assignment using a paper lottery system for randomization.

#### Sample size considerations for pilot field trial

As the study was a feasibility trial, a formal power calculation based on detecting evidence for effectiveness was not conducted. The feasibility RCT was done in a pragmatically chosen sample of 36 houses, with 24 receiving a floor (12 concrete, 12 low-cost floor) after baseline data collection and 12 not receiving a floor until the end of the study, when a new floor was also provided to these households. This sample was to primarily allow us to identify evidence of feasibility, and any problems with the intervention or research methods. Assuming sand fleas continue to develop in houses that did not receive a hard floor, we estimated approximately 80% of children in the control households would be infected at the end of the study. We expected hard floors to interrupt the developmental cycle of the sand fleas, however, we also assumed that some re-infection could take place elsewhere, i.e. in school or during family visits. With 12 houses (clusters) and 2 participants per house in each arm, we would have had 80% power to associate a 56% or higher reduction in infection, i.e. from 80% in the control to ≤35% in the intervention arm (assuming a coefficient of variation of 0.3). However, data from this pilot was meant to estimate the potential impact that can be expected and consequently use the data to estimate sample sizes for a large well-powered intervention trial.

#### Baseline assessments

A baseline survey was conducted in December 2019 comprising an interview with the household head or caregiver regarding the house structure and family members, education level, religion, sleeping arrangements in the house, animal ownership and whether animals entered the house, knowledge and practices of floor sealing techniques and floor care. At the same time the feet of all family members were carefully examined. For outcome measures, only infected children under the age of 17 years were enrolled. For infected children, the number of fleas were counted (all live, dead or manipulated) and clusters of fleas in which individual fleas are too close to each other to count were also counted.

The number of sites on both feet with acute symptoms (desquamation, fissures, ulcers, abscess,) were recorded as described previously [28]. To measure inflammation, the number of sites on the feet with infra-red hotspots were recorded using a FLIR One InfraRed adaptor for an android phone based on previous studies [29, 30].

#### Follow up assessments

Examination for flea counts and clinical symptoms were conducted at midline in May and endline in October 2020, five and 10 months after the floors had been installed. All family members found infected, were referred to the local community health worker network linked to the health facility for treatment. At the same time, the concrete and low-cost floors were observed for cracks and visible wear and tear, and load bearing, impact strength, resistance to abrasion and permeability test conducted in same way as for the slabs.

#### Satisfaction survey

Interviews were conducted with the head of household or the main caregiver following a structured questionnaire with closed and open style questions at the end of the study, to assess the experience, perceptions and acceptability of the new floors to the occupants. Questions explored if the family liked the floor and why, what changes it had made to their lives, their floor maintenance and cleaning routines and whether these had changed since receiving the floor.

#### Floor costs

All costs related to the installation of the floors were recorded in excel spreadsheets to estimate and compare the costs between the concrete floor and the new floor as installed during the project. All costs relating to the installation of each floor type were totalled and divided by the average house floor area to obtain an average cost per square metre of floor.

### Data analysis

#### Household interviews and focus group discussions

The responses to the baseline and endline household interviews were analysed semi-quantitatively, using frequencies for the closed questions. For open questions responses were grouped by question and then themes, and at endline by study arm.

The FGD analysis was conducted using the English transcripts. The transcripts were labelled according to groups and then merged by questions from the FGD guide. Analysis was conducted manually, identifying, and grouping themes for responses to each question.

#### Pilot field trial

The statistical package STATA v15 was used for all analyses. For disease outcomes, data were combined for analysis for the two study arms which received a floor, the concrete floor or the low-cost floor, so the study arms were ‘floor’ or ‘control’. Outcome measures that would be important in a large trial were explored. These include the proportion of infected participants in each study arm out of all participants that were infected at any time point and the infection intensity of participants in each household. The infection intensity was calculated for each participant as the sum of the number of embedded fleas plus the number of clusters multiplied by 5 (assuming a minimum of 5 fleas/cluster). We further explored the acute symptom score of the infected children in each household, generated by summing the number of sites (9 per foot) for each symptom; desquamation, fissures, ulcers, abscess and infra-red-hot spots with a maximum score of 18 for each symptom, leading to an overall maximum of 90.

Due to the small sample size, longitudinal analysis was not conducted. Outcome measures were compared between the two analysis arms, floor intervention or control at three timepoints separately, baseline during the dry season, midline at the height of the main rainy season and endline in the dry season. The association of the floor intervention with the proportion of infected participants was tested using mixed effects generalized logistic regression using the household ID number as a random effect. We explored the effect size expressed as an odds ratio with 95% confidence intervals to estimate the strength of the relationship between intervention and outcome.

Association with infection intensity, infra-red score and acute symptoms scores were explored using mixed effects generalized linear methods with a negative binomial distribution and log link function and using household ID as a random effect. The effect sizes are expressed as incidence rate ratios (IRR, exponentiated coefficients) with their 95% confidence interval.

Multivariable regression analysis for intervention impact and possible confounding covariates was conducted for infection intensity at endline. Firstly, bivariate analysis explored several possible covariates: age of child for most of the study, sex; subject’s relationship to household head; level of education of the household head; marital status of household head; religion of the family; income source; wall materials; roof materials; toilet type; ownership of animals; animals allowed inside house. Those with an association at a *p*-value less than 0.2 were then included in a multivariable model and backward elimination conducted.

## Results

### Focus group discussions

During discussions with community members, it became apparent that participants understood that smooth, hard floors play an important role in a house, from making it easier to keep clean, strengthening the house, adding aesthetic appeal to the house, and even preventing some diseases.

Female #1*: having hard floor keeps my house to be strong, F*emale #2: *it makes my house look good and clean and prevent some ants.* Female #*3: it also beautifies the house makes it easy to clean the house.* Male #1: *I think it prevent us from getting diseases or infections.*

Three traditional procedures of hardening floors were mentioned by participants in all of the six FGDs: (1) use of fire ash mixed with clay soil and water, (2) mixing clay soil with cow dung and termite mound soil or (3) actually building on top of a termite mound. However, it was also noted that only one family in the area is known to use cow dung for building. Participants mentioned that it is a method they have heard of being used by pastoralists such as the Maasai and Borana in other parts of the country. It was highlighted that cow dung is not used in the local cultural context as people feel it is unhygienic and think it would give the house a bad smell. It was also noted that the community used to build on top of old, abandoned termite mounds in the past when their settlements were further inland some decades ago. These days, termite mound soil is still occasionally used but not frequently since these mounds are not common in the study area. Now most households attempting to harden the floor are simply using what they call *“clay soil”*, sub-soil obtained from their own land or from that of their neighbours and mixed with water.

In response to the question why traditional methods such as the use of termite mound soil, ash and clay and cow dung mixtures are no longer used, respondents noted *“we are now in the digital world…also negligence has led us not to practice the traditional way of making floors, everybody (wants) good floors like tiles.* It was also mentioned that *“lack of knowledge and expertise has contributed”.* Regarding cow dung: *“it’s unhygienic and very dirty, the smell can make someone feel irritated”*.

When asked about who makes and cares for the floors, all respondents reported that it is mainly women and children who make the traditional clay soil floors. A few mentioned men may be involved, or someone may be employed to do it, or even a group of 3–5 people may work together to do it, depending on the size of the house. If a family pays an outsider to make the floor, the cost mentioned ranged from USD 2–10 (Kenya Shillings 200–1000) a day, but many mentioned that instead of paying in cash, they prefer to provide a special meal.

Males: *“the* (traditional) *floor is being made by women with the help of their children, one who has enough funds can employ someone to do it for him or her while in some communities they call for communal help”.*

Females: *“it also depends, some pay them on an exchange of a meal or cash basis for labour”.*

According to respondents, their *“clay soil”* floors need to be sprinkled with water 2–3 times a week to keep the dust down. After some time, cracks appear in the floor, and they need to be filled with more of the same clay soil and water mix. An extra layer of clay soil may be added onto the whole floor. All respondents reported that the floors quickly become dusty and must be swept 1–3 times a day depending on their condition to remove the dust. Over time this means that material is removed from the floor, lowering its level and undermining the walls, which in time causes them to collapse. One female youth mentioned that their dusty floors expose them to diseases.

*Males #1: “If the floor has stayed for so long you may now find some cracks. #3: If the floor was made of clay, you will find after some time it gets loose and become dusty. #6: Through continuous sweeping or cleaning it can weaken the house, which may lead the house to fall down”.*

*Female youth: “it becomes dusty when not watered and hardened which can cause diseases and infections”.*

### Floor development

All soil samples from the coastal villages could be described as almost entirely coarse-grained sand with a very low clay content. This included those soils the community referred to as a *“clay soil”* which they excavate from 1 m below the surface for construction of their house walls. This composition dictated the range of soil stabilization methods that could be tested in small slabs.

Due to the unavailability of a large enough covered enclosure for the slab tests in the study area, they had to be done outdoors. During the testing period the area experienced unusually high rainfall (Additional File 2 [21]) and flooding of some test slabs which likely prevented them from hardening properly, such as #1, 9 and 10 and led to the poor performance in the tests (Table 2).

**Table 2:** Outdoor floor slab test results and costs

	8-day RESULTS					28-day RESULTS						Costings				
Slab ID	Load bearing	Weight impact	Abrasion	water absorption	Total score	Load bearing	Weight impact	Abrasion	water absorption	Total score	overall score	Material costs (KES)	Labour (KES)	Total cost of slab (KES)	Total cost/m2 (KES)	Total cost/m2 (USD)^a^
1	1	3	2	3	9	1	3	1	2	7	16	2154	250	2404	1202	11.9
2	0	3	0	0	3	0	0	1	2	3	6	804	250	1054	527	5.2
3	0	3	0	0	3	0	1	1	0	2	5	804	250	1054	527	5.2
4	0	2	0	3	5	0	1	1	3	5	10	502	250	752	376	3.7
5	0	1	1	0	2	0	1	1	3	5	7	602	250	852	426	4.2
6	0	3	1	2	6	0	2	0	3	5	11	465	250	715	357	3.5
7	0	1	1	2	4	0	3	1	3	7	11	268	250	518	259	2.6
8A	0	2	1	3	6	0	1	1	3	5	11	402	250	652	326	3.2
8B	0	2	1	3	6	0	0	0	3	3	9	402	250	652	326	3.2
9	3	3	3	3	12	nd	nd	nd	nd	nd	nd	961	250	1211	605	6.0
10	3	3	3	3	12	nd	nd	nd	nd	nd	nd	1198	250	1448	724	7.2
11	0	2	1	1	4	0	1	1	1	3	7	134	250	384	192	1.9
12	0	2	1	3	6	0	1	1	3	5	11	134	250	384	192	1.9
13	0	3	1	1	5	0	2	1	3	6	11	483	250	733	366	3.6
14	0	1	1	3	5	0	0	1	3	4	9	268	250	518	259	2.6
15	0	1	1	2	4	0	3	1	3	7	11	1018	250	1268	634	6.3

^a^ Exchange rate Dec 2019 1 USD = 101.2 KES nd: not done

Most test slabs performed well in the load-bearing strength test, except slabs #9 and #10 which were made with silicate. A high weight impact chipped the surface of most floors but never cracked them. The abrasion tests left only slight marks on most floors except on slabs #2, #3 and #4. Most of the slabs absorbed at least some of the water after 10 minutes, except for slabs #2 and #3. Two test slabs reached the maximum test score of 12 on the scale (i.e. the worst) at the 8-day tests, slabs #9 and #10, which both did not contain any cement for stabilisation (Table 1). These two slabs were not tested again. The lowest test scores indicate the best floors, which were slabs #2, #3, #5, and #11 all with overall scores after 28 days below 8. However, the material cost estimate of USD 2/ m^2^ was the lowest for slab #11 so the study team selected the composition of slab #11 to go forward in the pilot field trial. This slab had a base layer of 10 cm and a floor layer of 5 cm and comprised of local subsoil mixed with cement in a 1:9 ratio.

### Pilot field trial

#### Floor installation

The floors were installed by two teams each comprising one trained mason and one labourer, one team installing concrete floors and the other installing the low-cost floor. For some of those households who had a young man in the family, he would also assist with the manual labour, but this was not required of them. Families provided all the water that was needed. The concrete floors took 1 a half to 2 days to complete while the low-cost floor took only 1 day for the small house size of around 30 m^2^. Although families were asked to empty all belonging from the houses ahead of time, often they had not done so when the masons arrived, delaying the floor installation. Families usually had other family members living nearby with whom they could stay and store items while they had to be out of their own house. A major challenge was families not complying with the daily water sprinkling and the instruction to stay out of the house for 7 days. Although routine checks were not included in the protocol, occasional spot checks revealed most families returned to the house after 3 days.

#### Characteristics of households and retention of participants

**Fig. 3:**
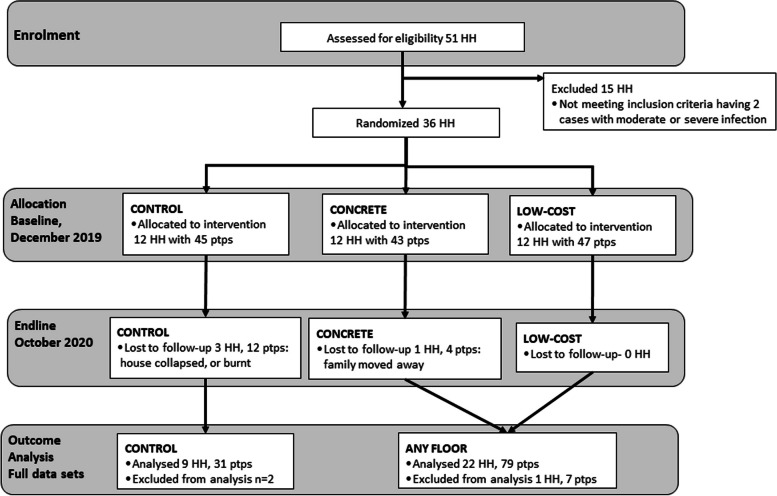
Study Flow Chart. HH = household, ptps = participants, infecte*d children enrolled at baseline*

A total of 36 households, occupied by 69 adults (over 17 years) and 141 children, were enrolled and randomized to the three study arms as illustrated in the study flow chart (Fig. 3). Seven of the 36 enrolled houses were improved semi-permanent houses having stone brick walls with cement and an iron sheet roof, but with an unsealed earthen floor. Four of these seven houses received a floor. The other 29 houses had walls of either palm leaf, mud in a wood frame or mixed mud and rough stones in a wood frame combined with either a palm leaf or iron sheet roof as previously illustrated in Fig. 1. Only children aged 17 years and under who were infected at baseline, were enrolled as participants for follow-up evaluation of outcome measures. A total of 135 infected children were enrolled at baseline, ranging between 2 and 5 per household (median 4, IQR 3–4). There were 12 households with 45 infected children in the control group and 24 households with 90 infected children in the intervention groups. Over the course of the study, four (11%) complete households with 16 participants were lost to follow up due to either the house falling or burning down (*n* = 3), or the family moving away (*n* = 1). For the analysis a further 1 household and a total of 9 participants were excluded from the analyses due to having incomplete data sets. The final full data sets for analyses included 9 households with 31 children in the control group and 22 households with 79 children in the intervention group (Fig. 3). Of the 110 children in the analyses, 57% were boys and the average age was 7 years (SD 2.0, range 3 to 12 years).

At baseline, the household head or caregiver in each participating family was asked what they felt about the floor they currently had. Most of them (*n* = 32 of 36) said they did not like the earthen floors as; “*It is dusty and not pleasant”*, “*Has dust while sweeping”*. A few (*n* = 6) associated these floors with tungiasis infection “*Floor is dusty which I believe brings jiggers*”, others with flies (*n* = 5) “*It is dusty and loose which brings flies*” or respiratory illness (n = 3); “*Has a lot of dust which causes flu*”. Like the participants in the FGDs, the majority of household heads (*n* = 22) in the trail reported to reduce the dust and harden the floors by pouring water on them daily. Only four said they smeared mud to harden the floor occasionally, and none had used cow-dung. For those who had never tried to harden their floor they stated this was because they couldn’t afford a better floor, “*I don’t have money to get a good floor*”; did not have the knowledge or skills, “*I have no skills to do it*”; or they did not have time to do it, “*Am always out for work, no time to harden the floor*”. All the households had attempted to control the tungiasis in their family by either spraying the house floor with water alone (*n* = 5) or mixing water with neem (*Azadirachta indica*) leaf extract (*n* = 12) or paraffin (*n* = 2), being locally practiced interventions believed to kill or prevent fleas in the soil.

#### Tungiasis infection

At baseline, all enrolled participants were infected according to selection criteria, hence the proportion of participants (prevalence) who were infected was 100%. By midline, the prevalence decreased in both the control and the flooring groups, to 65% in the control group and 47% in the floor group (Table 3). By the end of the study, 10 months after floor installation, the prevalence increased again in both study arms to 71% in the control arm and 54% among those with a floor. While the odds of infection among children in the intervention arm was half that for children in the control arm at midline (OR 0.46) and endline (OR 0.49), this did not reach statistical significance at the 0.05 level since the pilot was not sufficiently powered (Table 3).

**Table 3:** Bivariable model for proportion of participants infected by study arm and time point

Time point	Study arm	Percent	95% CI^1^		OR^2^	95% CI^1^		p
Baseline	Control	100						
	With floors	100						
Midline	Control	65	0.46	0.79	1			
	With floors	47	0.36	0.58	0.46	0.12	1.59	0.241
Endline	Control	71	0.53	0.84	1			
	With floors	54	0.43	0.65	0.49	0.11	1.86	0.320

^**1**^ confidence interval ^2^ odds ratio

#### Infection intensity and associated symptoms

Infection intensity as well as the associated symptoms as measured by infrared and acute morbidity score were similar at baseline for children in the two study arms (Table 4). By midline, the infected participants’ infection intensity and symptoms had decreased considerably in both study arms. However, for all three outcomes, the reduction in the flooring group was around 40% higher than in the control group, as expressed by the effect sizes in Table 4. At endline, the median infection intensity was back to baseline levels among the control households (median 18 fleas, IQR 9–28) but remained low among participants living in a house with improved a floor (median 8 fleas, IQR 2–14, Table 4). Median infection intensity, median inflammation and acute symptom scores for children in the floor groups were over 50% lower than for those in the control group at endline. Whilst this did not reach statistical significance at the 0.05 level in the bivariate analysis, it did when adjusted for confounders (aIRR 0.53, 95% CI 0.29–0.97, Table 5). There were no interactions between any of the independent variables.

**Table 4:** Bivariable model for Household median infection intensity, infra-red and acute morbidity for cases by study arm and time point

			Infection intensity		Infra-red score		Acute morbidity	
Time point	Study arm	N^1^	Median (IQR^2^)	IRR^3^ (95% CI^4^)	Median (IQR)	IRR (95% CI)	Median (IQR)	IRR (95% CI)
Baseline	Control	31	18 (7–28)	1	6.5 (4–9)	1	13 (8–17)	1
	Floors	79	14 (7–29)	0.90 (0.54–1.50)	6 (4–9)	1.07 (0.8–1.45)	13 (8–20)	1.07 (0.79–1.44)
Midline	Control	20	5 (2–12.5)	1	4.5 (1–7.5)	1	7 (3–10.5)	1
	Floors	36	3.5 (2–8)	0.66 (0.29–1.49)	2 (1–5)	0.59 (0.26–1.37)	3 (1–7.5)	0.55 (0.22–1.39)
Endline	Control	22	18 (9–28)	1	4 (2–5)	1	8.5 (4–14)	1
	Floors	43	8 (2–14)	0.47 (0.20–1.12)	2 (0–4)	0.47 (0.18–1.23)	4 (1–8)	0.43 (0.15–1.21)

^1^ N: number of participants. ^2^ interquartile range ^3^IRR: incidence rate ratio ^4^ Confidence interval

**Table 5:** Bivariable and multivariable regression for infection intensity among cases at the end of the study

			Bivariable		Multivariable	
		N^1^	IRR^2^ (95% CI^3^)	P	aIRR^4^ (95% CI)	P
Intervention group	control	31	1		1	
	floor	79	0.47 (0.20–1.12)	0.089	0.53 (0.29–0.97)	0.039
Sex	female	49	1			
	male	61	1.01 (0.69–1.47)	0.961		
Age^5^		110	1.00 (0.94–1.06)	0.904		
Relationship to HHH^6^	child	80	1		1	
	grandchild	24	1.38 (0.66–2.89)	0.393	1.14 (0.57–2.30)	0.705
HHH education level	complete primary	15	1		1	
	incomplete primary	68	1.15 (0.33–3.93)	0.828	0.28 (0.09–0.90)	0.032
	none	27	1.38 (0.37–5.07)	0.629	0.99 (0.38–2.55)	0.981
Religion	christian	39	1		1	
	muslim	6	0.09 (0.01–0.98)	0.048	0.16 (0.02–1.25)	0.082
	traditionist	58	1.18 (0.52–2.71)	0.687	1.45 (0.79–2.66)	0.235
Income source	employed	25	1			
	casual labour	38	1.00 (0.31–3.25)	0.998		
	self employed	26	0.97 (0.27–3.51)	0.966		
	selling alcohol	10	1.11 (0.25–4.90)	0.892		
	none	11	1.07 (0.24–4.87)	0.928		
HHH Marital status	married	84	1			
	single	26	0.99 (0.35–2.75)	0.977		
Wall material	stone blocks^7^	32	1			
	mud and stone^8^	26	0.53 (0.21–1.39)	0.197		
	mud	41	0.32 (0.12–0.84)	0.021		
	palm leaf	11	0.26 (0.07–0.94)	0.04		
Roof materials	iron sheet	53	1			
	palm leaf	57	0.40 (0.19–0.87)	0.021		
Water source	own tap	9	1			
	community tap	91	0.93 (0.21–4.09)	0.92		
	shared well/ borehole	10	1.02 (0.13–7.97)	0.986		
Toilet type	open defecation	51	1		1	
	traditional latrine	59	2.42 (1.09–5.40)	0.03	5.01 (1.98–12.67)	0.001

^1^ N: number of participants. ^2^ IRR: incidence rate ratio ^3^ Confidence interval ^4^ aIRR: adjusted incidence rate ratio ^5^ age in years most surveys ^6^HHH: head of household ^7^ stone blocks cut from local coral rock ^8^ rough coral stones

#### Floor durability and costs

By the end of the study, 12 of 12 low-cost floors had developed fine lines in the finish and one had a larger open crack. Of the concrete floors, 10 out of 12 also had fine lines in the finish. Most fine cracks were observed around the edges of the floor where it joined the wall, particularly in houses where the residents had replaced or repaired the walls. From the four tests conducted on the floors, neither the 100 kg weight nor the weight drop had any impact on either the concrete or the low-cost floor (Additional file 2). However, some of the new floors were impacted slightly by the abrasion test (median score 1) and seemed to absorb some water in the permeability test (median score of 2).

The average house size in the study was 30 m^2^, ranging from 8 m^2^ to 94 m^2^. The only costs for the new low-cost floor were for cement, transport of materials and workmen and the local labour charges since the sub-soil was taken directly from the plot. The total cost of installing the soil-stabilised floor for the average house size was US$115 ($3.9/m^2^) (Table 6). For the concrete floor there was the additional cost of ballast, hardcore and river sand and transporting these with a truck from a local supplier. The concrete floors for the average house size cost a total ofUS$278, an average of US$9.3/ m^2^.

**Table 6:** Costs for the installation of the floors in an average house of 30m^2^ during the pilot study. (Exchange rate at the time, December 2019 0.0097)

	Item	Units	No. of Units	Unit Cost US$	Total Cost US$
Low-cost floor	Cement	bag	6	6.3	37.8
	Transport	day	1	9.7	9.7
	Labour	pax	2	34.0	67.9
	Total				115.4
	Total per m^2^				3.9
Local concrete floor	Cement	bag	8	6.3	50.4
	River sand	tonne	4	17.5	69.8
	Ballast	tonne	2	24.3	48.5
	Hardcore	tonne	2	14.6	21.8
	Transport	day	2	9.7	19.4
	Labour	pax	2	34.0	67.9
	Total				277.9
	Total per m^2^				9.3

#### Community perception of improved floors

At the end of the study one adult from each of 32 houses that participated till the end, was asked their opinion about their new floor (11 with concrete floor and 12 with a low-cost floor). Everyone with the concrete floor (11) and 10 of those with the low-cost floor (10/12) said they were satisfied with it. The two households who indicated that they did not like the low-cost floor reasoned that “*some part of the living room* [have] *some cracks”* and *“in the sitting room the surface is flacking…there is small hole which allows some ants penetrate through and have some cracks”.*

Those who received the concrete and low-cost floor had very similar reasons for liking the floor. The most common reasons being it was much easier to clean (13 of 20), reduced dust (11 of 20), and there were less insects in the house, particularly ants and fleas (9 of 20). *“I like it because it reduces the amount of fleas, it’s easy to sweep and clean and less dust in the house”; “my house has no more jiggers”.*

Other reasons for approving of the floor were ability to sleep and sit on the floor without getting dirty and aesthetics; *“my kids are not getting dirty anymore”; “my children can now sit down on the floor and study”; “it makes my house very clean and smart”.* One person also felt the floor protects from respiratory illness *“kids are not infected with flu”.*

Some respondents from houses with the concrete floor, but none from houses with the low-cost floor mentioned that it made their house stronger (6 of 11 houses): *“it is long lasting it has strengthened the house”; “it has made my house firm”*.

## Discussion

Through a formative co-creation process and working with a local CBO in coastal Kenya, this study set out to understand the local practices in respect to improving house floors; to assess the feasibility of creating a low-cost floor with local material, and to pilot the low-cost floor to gain insight into potential challenges and initial indication of factors that might be associated with the impact on disease outcomes. We found that rural homeowners do understand that having a dust-free, washable floor has a positive impact on health. The major constraint for not improving floors was the perceived financial burden that was associated with standard concrete floors. The only improved floor known to these communities is a complex, multi-layered concrete floor requiring materials transported from a distance and qualified, experienced masons to install them. Masons charge more than US $10 a day for their services and they would be needed for several days to lay the concrete floor. Consequently, even households, that had improved their houses with coral block walls and iron sheet roofs, had made no effort to seal the floor. Very few people, including local masons, have considered a simpler, cheaper alternative which would make sealing a floor more achievable, yet traditional rural houses have no need for the high load bearing provided by the concrete floors.

One limitation of the study was the necessity to conduct the slab trials outdoors since no covered area was available. As we explored different materials for flooring, using outdoor slab trials, we received some of the heaviest rainfall ever experienced in the area [21] (additional file 2). Many of the different slabs were badly damaged by flooding, including those with cow-dung which never had a chance to harden. We expect this biased our results to using cement as a soil stabilizer in the end, since it requires to be kept wet for several days after laying to complete the chemical curing process and properly harden the mixture. Other material combinations, such as cow dung, plant fibre and fire ash might however be good options in drier conditions and should be further explored in areas where these are frequently used and available. Adding cow-dung to soil for adobe brick making has been shown in other studies to bind soil particles, increase the strength of bricks and decrease water absorption [24].

By incorporating cement as the binder, the floor composition selected had the benefit of only using locally available materials, cement being widely available in the study area as well as across Kenya. The cost for the new floor was one third that of the concrete floors installed as part of the trial. This might already be affordable for many rural families if demand (priority) is created through communication and education campaigns, but families at the lowest socio-economic level might still depend on private sponsors, charitable organizations and other donors wishing to support low-income households with sustainable disease prevention measures; such organizations might find the soil-stabilised floor cost-effective. In fact, both the masons privately, and the involved CBO have since been hired to install the low-cost floor for other families in the local area and further away, some funded by charitable organizations as they attempt to assist communities with tungiasis.

While we appreciate 10 months is a short time to evaluate a building intervention, this was a study with a limited budget and the main aim was to assess the factors affecting the feasibility of retrofitting floors and assess potential impact for the planning of larger and longer trials. By the end of the pilot trial, most of the floors were still intact, in a condition that could be easily swept and washed to maintain good hygiene standards. The common minor fractures in the superficial finish were likely a result of the high water content of the slurry as well as not sufficiently curing the floor and returning to live in the house before the 7 day limit, so that it dried out too quickly and was exposed to stress too early. Any future project might consider a different finish layer, which is less prone to cracking and would need to include communication and spot-check strategies to achieve compliance with floor care post-installation by the homeowners. The training and supervision of the local masons was not very extensive in this pilot trial and based on field records, it became apparent that there were issues with the masons complying with required timelines and standard procedures for making the soil-stabilized floors. This may have contributed to some of the damage observed. In a larger trial investigating health impact, it will be necessary to develop procedures that are easily understood and invest more time for hands-on training and supervision of masons and their labourers to ensure all floors are of the same quality.

In terms of disease outcomes, the pilot trial showed promise for success for a definitive trial, despite the multitude of operational challenges that were experienced during the implementation of this study. Overall, the results suggest that the odds of infection could be reduced by around 40–50% for people living in a house with improved floor, as compared to those having a natural floor. Similarly, infection intensity and associated acute symptoms were reduced when compared to the control groups. The latter is especially important, as it is the goal of any control programme to reduce disease-induced morbidity. By reducing infection and inflammation, a floor intervention can be expected to reduce the pain and extreme itching associated with tungiasis which disturbs people’s sleep, ability to walk and concentrate in school or at work [11] which should be assessed as secondary outcomes in a definitive RCT.

The outcomes of the pilot trial may have been affected by two major unforeseen events. The study location is usually arid, with very little rainfall and observations on disease outcomes were planned to be evaluated largely during the dry season. However, there was an unusual climate event over the first 8 months of the observation period with high and consistent rainfall throughout (supplementary materials). A previous study from Brazil demonstrated that the prevalence of tungiasis was associated with rainfall, being lowest during and immediately following the wet season and highest in the dry season [20]. Anecdotal reports in Kenya also suggest a similar seasonal pattern which might in part explain the drop in disease outcomes in both the control and intervention houses at midline.

Furthermore, the trial coincided with the start of the COVID-19 pandemic, which in Kenya resulted in restrictions and school closure from March 2020, 3 months into the trial, for the rest of the year. This affected the behavioural patterns of household residents and especially those of the children. Based on the household questionnaires, the children participating in the trial often spent time away from home visiting friends or family, with several reporting to sleep at different locations at the end of the trial than at the start. These variable behaviours might have resulted in some children being re-infected even when there was a floor in their house. Since family behaviours may impact how effective a flooring intervention can be, there is need to implement more formative research to understand how families interact with their house floors, including hygiene behaviours and general cleanliness.

We witnessed during spot-check visits, that mud walls shed a significant amount of soil and dust onto the floors and that soil was being brought into the house on feet and shoes. Also, faeces from chicken were frequently observed indoors. Furthermore, due to the absence of cupboards and shelves, personal items were covering large floor areas, making it difficult to keep clean. Not all families adopted a cleaning routine after having received a new floor, which might also have contributed to not fully interrupting the transmission cycle inside houses with improved floors. These observations indicate that a flooring intervention should be coupled with targeted behaviour change communications to increase the success of interrupting disease transmission.

Nevertheless, we observed that un-improved flooring was associated with a resurgence of the infection intensity and morbidity to baseline levels at endline during the peak of the dry season that started 3 months earlier. This resurgence was not seen among participants from the households with improved floors. Data from the trial hold promise that low-cost flooring interventions can in fact reduce disease prevalence and infection intensity. Based on the pilot data, where we have seen a disease prevalence at endline of 71% in control houses and 54% in intervention houses, sample size estimates suggest we would require 83 houses with a minimum of two tungiasis infected participants per house, per study arm (83 with floor and 83 without floor; total 166 houses) to associate the difference with the intervention with 80% power at a 95% confidence level. In our study, whole households were lost to follow up either due to families moving away or due to the makeshift houses collapsing or burning down. Overall, we lost 14% of the households and 19% of the participants to follow-up. Hence, in a fully powered trial there is need to recruit at least 20% more households and participants than needed. Whilst the collapse of the house in our trial happened several months after the installation of the floor and is most likely not associated with it, it might be advisable for a larger trial, to have a minimum set of indicators for structural integrity, as inclusion or exclusion criteria.

## Conclusions

This study provided proof of principle that the development of low-cost, locally appropriate soil-stabilised floors is possible using a community-based co-creation approach and that retrofitting those in existing traditional, rural houses is feasible. A simple flooring solution might well be within the financial reach of rural homeowners, and it might only require training of local masons and local action groups to promote such floors. These would need to be combined with targeted behaviour change campaigns to help the community to understand the importance of sealing a house floor and keeping it clean to prevent diseases. Such an intervention would not only affect the development of the sandfleas that cause tungiasis, but would likely also reduce other soil-transmitted parasites and bacteria associated with diarrheal disease [31, 32], hence providing a promising tool for integrated disease prevention at the intersection of the built environment and health. The impact on tungiasis has shown promise in this feasibility study to warrant the recommendation of a definitive RCT to provide evidence for decision makers to integrate this intervention in programs run by governments or non-governmental organizations.

### Supplementary Information

**Additional file 1.**

**Additional file 2.**

**Additional file 3.**

## Data Availability

All data analysed during this study are included in this published article and its supplementary information files (Additional File 3).

## Abbreviations

CBO: community-based organisation
FGD: focussed group discussion
IQR: interquartile range
KES: Kenya shillings
NTD: neglected tropical diseases
SD: standard deviation
US$: united states dollar
WHO: world health organization

## References

[1] Feldmeier H, Heukelbach J, Ugbomoiko US, Sentongo E, Mbabazi P, von Samson-Himmelstjerna G (2014). Tungiasis--a neglected disease with many challenges for global public health. PLoS Negl Trop Dis..

[2] Elson L, Wright K, Swift J, Feldmeier H. Control of Tungiasis in absence of a roadmap: grassroots and global approaches. Trop Med Infect Dis. 2017;2(3) 10.3390/tropicalmed2030033. 10.3390/tropicalmed2030033 PMC6082108 30270889

[3] World Health Organization (2021). Neglected Tropical Diseases.

[4] Eisele M, Heukelbach J, Van Marck E, Mehlhorn H, Meckes O, Franck S (2003). Investigations on the biology, epidemiology, pathology and control of Tunga penetrans in Brazil: I. Natural history of tungiasis in man. Parasitol Res..

[5] Nagy N, Abari E, D'Haese J, Calheiros C, Heukelbach J, Mencke N (2007). Investigations on the life cycle and morphology of Tunga penetrans in Brazil. Parasitol Res..

[6] Heukelbach J, de Oliveira FA, Hesse G, Feldmeier H (2001). Tungiasis: a neglected health problem of poor communities. Tropical Med Int Health..

[7] Ugbomoiko US, Ifeanyi Ofoezie E, Heukelbach J (2007). Tungiasis: high prevalence, parasite load, and morbidity in arural community in Lagos state. Nigeria Int J Dermatol..

[8] Wilcke T, Heukelbach J, Cesar Saboia Moura R, Regina Sansigolo Kerr-Pontes L, Feldmeier H (2002). High prevalence of tungiasis in a poor neighbourhood in Fortaleza, Northeast Brazil. Acta Trop..

[9] Feldmeier H, Eisele M, Van Marck E, Mehlhorn H, Ribeiro R, Heukelbach J (2004). Investigations on the biology, epidemiology, pathology and control of Tunga penetrans in Brazil: IV. Clin Histopathol Parasitol Res..

[10] Feldmeier H, Eisele M, Saboia-Moura RC, Heukelbach J (2003). Severe tungiasis in underprivileged communities: case series from Brazil. Emerg Infect Dis..

[11] Wiese S, Elson L, Feldmeier H (2018). Tungiasis-related life quality impairment in children living in rural Kenya. PLoS Negl Trop Dis..

[12] Feldmeier H, Heukelbach J, Eisele M, Sousa AQ, Barbosa LM, Carvalho CB (2002). Bacterial superinfection in human tungiasis. Tropical Med Int Health..

[13] Muehlen M, Feldmeier H, Wilcke T, Winter B, Heukelbach J (2006). Identifying risk factors for tungiasis and heavy infestation in a resource-poor community in Northeast Brazil. Trans R Soc Trop Med Hyg..

[14] Ugbomoiko US, Ariza L, Ofoezie IE, Heukelbach J (2007). Risk factors for tungiasis in Nigeria: identification of targets for effective intervention. PLoS Negl Trop Dis..

[15] Mwangi J, Ozwara H, Gicheru M (2015). Epidemiology of tunga penetrans infestation in selected areas in Kiharu constituency, Murang’a county, Kenya. Trop Disease Travel Med Vaccines..

[16] Wiese S, Elson L, Reichert F, Mambo B, Feldmeier H (2017). Prevalence, intensity and risk factors of tungiasis in Kilifi County, Kenya: I. Results from a community-based study. PLoS Negl Trop Dis..

[17] Elson L, Wiese S, Feldmeier H, Fillinger U (2019). Prevalence, intensity and risk factors of tungiasis in Kilifi County, Kenya II: results from a school-based observational study. PLoS Negl Trop Dis..

[18] Centers for Disease Control and Prevention (2020). How Fleas Spread Disease.

[19] a4architect (2013). Analysis of the cost of construction of a concrete slab in Kenya.

[20] Heukelbach J, Wilcke T, Harms G, Feldmeier H (2005). Seasonal variation of tungiasis in an endemic community. Am J Trop Med Hyg..

[21] World Weather Online (2022). World Weather Averages.

[22] Felix F, Udoeyo AOC, Jajere S. Mound Soil as Construction Material. J Mater Civil Eng. 2020;12(3) 10.1061/(ASCE)0899-1561(2000)12:3(205).

[23] Bagampadde U, Kaddu D, Hawumba JF, Ntale M (2023). An experimental termite enzyme-based stabilizer for treating aged pavement laterites. Int J Pavement Res Technol..

[24] Millogo Y, Aubert J-E, Séré AD, Fabbri A, Morel J-C (2016). Earth blocks stabilized by cow-dung. Mater Struct..

[25] Okogbue C. Stabilization of clay using Woodash. J Materials Civil Eng. 2007;19 10.1061/(ASCE)0899-1561(2007)19:1(14).

[26] Nath BD, Sarkar G, Siddiqua S, Rokunuzzaman M, Islam MR (2018). Geotechnical properties of wood ash-based composite fine-grained soil. Adv Civil Eng..

[27] Consoli Nilo C, Daassi-Gli Cocou Auxence P, Ruver Cesar A, Lotero A, Scheuermann Filho Hugo C, Moncaleano Cindy J (2021). Lime–Ground Glass–Sodium Hydroxide as an Enhanced Sustainable Binder Stabilizing Silica Sand. J Geotech Geoenviron..

[28] Kehr JD, Heukelbach J, Mehlhorn H, Feldmeier H (2007). Morbidity assessment in sand flea disease (tungiasis). Parasitol Res..

[29] Schuster A, Thielecke M, Raharimanga V, Ramarokoto CE, Rogier C, Krantz I (2017). High-resolution infrared thermography: a new tool to assess tungiasis-associated inflammation of the skin. Trop Med Health..

[30] Elson L, Matharu AK, Riithi N, Ouma P, Mutebi F, Feldmeier H (2023). Characterization of tungiasis infection and morbidity using thermography in Kenya revealed higher disease burden during COVID-19 school closures. Infect Dis Poverty..

[31] Exum NG, Olórtegui MP, Yori PP, Davis MF, Heaney CD, Kosek M (2016). Floors and toilets: Association of Floors and Sanitation Practices with fecal contamination in Peruvian Amazon Peri-urban households. Environ Sci Technol..

[32] Steinbaum L, Njenga SM, Kihara J, Boehm AB, Davis J, Null C (2016). Soil-transmitted helminth eggs are present in soil at multiple locations within households in rural Kenya. PLoS One..

