# Supplementary material for: Developing low-cost house floors to control tungiasis in Kenya – a feasibility study
Source: BMC Public Health. 2023 Dec 12;23:2483. doi: 10.1186/s12889-023-17427-4 (PMC10714545; doi:10.1186/s12889-023-17427-4)

**Elson et al. Developing low-cost house floors to control tungiasis in Kenya – a feasibility study**

**Additional file 2:**

**Figure: Rainfall data for the study location showing unusual weatehr events during the trial period.**


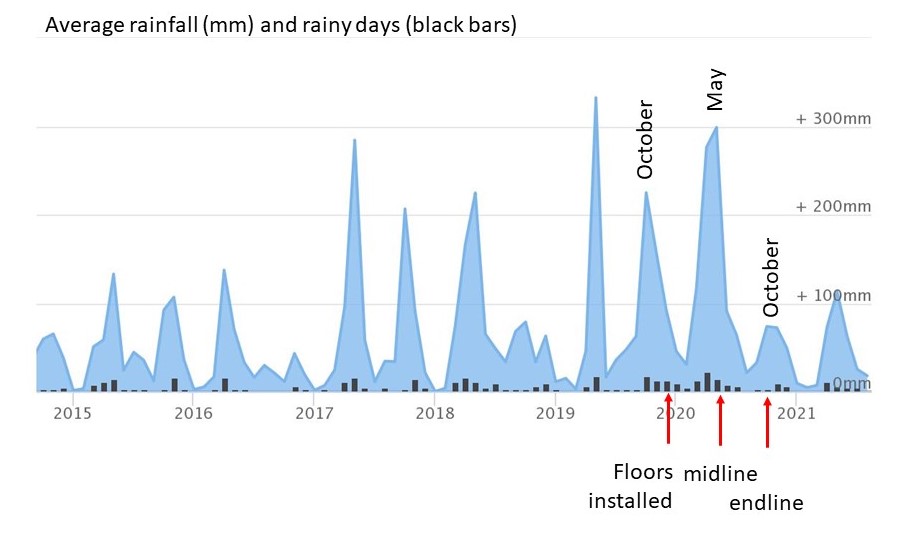

Supplement: Supplementary file 1 — Additional file 1. [file 12889_2023_17427_MOESM1_ESM.docx]
