# Supplementary material for: Developing low-cost house floors to control tungiasis in Kenya – a feasibility study
Source: BMC Public Health. 2023 Dec 12;23:2483. doi: 10.1186/s12889-023-17427-4 (PMC10714545; doi:10.1186/s12889-023-17427-4)
Supplement: Supplementary file 2 — Additional file 2. [file 12889_2023_17427_MOESM2_ESM.docx]

## **Elson et al. - Developing low-cost house floors to control tungiasis in Kenya – a feasibility study**

## **Additional File 3**

**Table: Test scores for floor durability and performance during pilot survey**

|  |  | **Four months** | | | | **End of study** | | | |
| --- | --- | --- | --- | --- | --- | --- | --- | --- | --- |
| HH_Study_ID | Floor_type | compression | abrasion | impact | permeable | compression | abrasion | impact | permeable |
| 1 | Concrete | 0 | 0 | 0 | 2 | 0 | 0 | 0 | 1 |
| 10 | Concrete | 0 | 0 | 0 | 2 | 0 | 0 | 0 | 1 |
| 11 | Concrete | 0 | 0 | 0 | 2 | 0 | 0 | 0 | 1 |
| 12 | Concrete | 0 | 0 | 0 | 2 | 0 | 0 | 0 | 0 |
| 14 | Concrete | 0 | 0 | 0 | 3 | 0 | 0 | 0 | 0 |
| 16 | Concrete | 0 | 0 | 0 | 2 | 0 | 0 | 0 | 1 |
| 15 | Concrete |  |  |  |  | 0 | 0 | 0 | 2 |
| 22 | Concrete | 0 | 0 | 0 | 1 | 0 | 0 | 0 | 1 |
| 26 | Concrete | 0 | 0 | 0 | 2 | 0 | 0 | 0 | 0 |
| 31 | Concrete | 0 | 0 | 0 | 2 |  |  |  |  |
|  | **Median** | **0** | **0** | **0** | **2** | **0** | **0** | **0** | **1** |
|  |  |  |  |  |  |  |  |  |  |
| 2 | Low-cost floor | 0 | 1 | 0 | 2 | 0 | 0 | 0 | 1 |
| 5 | Low-cost floor | 0 | 1 | 0 | 2 | 0 | 0 | 0 | 2 |
| 9 | Low-cost floor |  |  |  |  | 0 | 1 | 0 | 2 |
| 13 | Low-cost floor | 0 | 0 | 0 | 3 | 0 | 0 | 0 | 2 |
| 18 | Low-cost floor | 0 | 1 | 0 | 2 | 0 | 1 | 0 | 2 |
| 20 | Low-cost floor | 0 | 0 | 0 | 0 |  |  |  |  |
| 21 | Low-cost floor | 0 | 0 | 0 | 2 | 0 | 1 | 0 | 2 |
| 25 | Low-cost floor | 0 | 1 | 0 | 2 | 0 | 1 | 0 | 3 |
| 27 | Low-cost floor | 0 | 1 | 0 | 1 | 0 | 1 | 0 | 2 |
| 30 | Low-cost floor | 0 | 1 | 0 | 2 | 0 | 1 | 0 | 2 |
| 32 | Low-cost floor | 0 | 1 | 0 | 2 | 0 | 1 | 0 | 2 |
| 34 | Low-cost floor | 0 | 0 | 0 | 2 | 0 | 1 | 0 | 2 |
|  | **Median** | **0** | **1** | **0** | **2** | **0** | **1** | **0** | **2** |
